# Maleimide availability in thiol-maleimide peptide conjugation on PEGylated liposomes: a reactive-site accounting framework illustrated using hepatic drug-delivery systems

**DOI:** 10.3389/fddev.2026.1964437

**Published:** 2026-10-13

**Authors:** Kashif Maroof, Rükan Genç

**Affiliations:** 1 SUNUM, Sabanci University Nanotechnology Research Centre, Sabanci University, Istanbul, Türkiye; 2 Department of Chemical Engineering Faculty of Engineering Mersin University, Mersin, Türkiye

**Keywords:** conjugation efficiency, DSPE-PEG-maleimide, hepatic nanomedicine, hepatocellular carcinoma, maleimide availability, peptide-targeted liposomes, thiol-maleimide conjugation

## Abstract

Peptide-targeted PEGylated liposomes are a widely investigated active-targeting strategy for hepatocellular carcinoma (HCC) and other hepatic indications. A critical but underreported formulation variable is effective maleimide availability: the number of outward-facing, unhydrolysed, sterically accessible maleimide groups present when peptide thiols are coupled. In the seven hepatic systems examined, authors often report nominal lipid or peptide input without measuring reactive sites, conjugation efficiency, or final surface density. Hydrolysis, steric access, bilayer orientation, and peptide-thiol oxidation are individually well-established features of thiol-maleimide chemistry; the contribution of this Perspective is not to identify these phenomena but to integrate them into a quantitative accounting framework that links nominal input to biological outcome. We therefore propose a reactive-site accounting framework that separates six distinct quantities often conflated in practice: nominal maleimide input (the formulation’s stated lipid composition), reactive maleimide (the outward-facing, unhydrolyzed fraction actually present at coupling), available peptide thiol (the free-thiol fraction remaining after synthesis, reduction or thiolation, and handling), conjugation efficiency (the proportion of input peptide that is actually conjugated), final conjugated ligand density (molecules per particle), and biologically accessible ligand density (the fraction capable of engaging its receptor in the biological milieu). Route selection, such as distinguishing maleimide-lipid pre-incorporation, post-insertion of a lipid conjugate, and direct coupling on preformed liposomes, should be evaluated experimentally rather than assumed. The chemical limitations underlying this framework are not specific to hepatic targeting; the seven hepatic systems examined here serve as an illustrative case set for a broader reporting problem in thiol-maleimide functionalization of nanocarriers. Reporting these measures would make density–response relationships easier to interpret and peptide-liposome studies, hepatic and otherwise, easier to reproduce.

## Introduction

Hepatocellular carcinoma (HCC) remains one of the most lethal malignancies worldwide, and the liver is among the most extensively targeted organs in nanomedicine (Shaker et al., 2025). Differential receptor expression across hepatocytes, hepatic stellate cells, sinusoidal endothelial cells, Kupffer cells, and malignant hepatocytes provides multiple opportunities for cell-selective delivery beyond passive EPR-driven accumulation (Maroof et al., 2026). Accordingly, actively targeted liposomal systems have been designed by decorating their surface with ligands such as antibodies, antibody fragments, aptamers, carbohydrates, small molecules, and peptides that recognize receptors overexpressed in HCC or in the tumor microenvironment. These ligands are commonly introduced through post-insertion into preformed liposomes or by covalent conjugation to functionalized lipids, most often PEGylated lipid anchors bearing reactive groups such as maleimide, NHS ester, carboxyl, azide, or biotin–streptavidin handles. Among these strategies, peptide-decorated PEGylated liposomes are particularly attractive because peptides offer small size, tunable affinity, relatively straightforward synthesis, and compatibility with site-specific conjugation chemistries, enabling receptor-directed accumulation while preserving the stealth and stability advantages of PEGylated liposomal carriers (Maroof et al., 2026).

The reported peptide-targeting strategies for HCC and liver-associated cells are now diverse, both in terms of receptor selection and conjugation chemistry (Maroof et al., 2026). Cyclic RGD has been conjugated through maleimide–PEG–DOPE to target activated hepatic stellate cells via collagen VI receptor-mediated endocytosis, resulting in approximately 10-fold higher stellate-cell accumulation than unmodified liposomes (Du et al., 2007). GE11, an EGFR-targeting dodecapeptide, has similarly been introduced through DSPE–PEG–maleimide to improve HCC tumor accumulation of liposomes co-delivering curcumin and paclitaxel (Tang et al., 2024). BR2, a cancer-selective cell-penetrating peptide that exploits ganglioside overexpression in HCC, has been conjugated to DSPE–PEG–maleimide for intracellular cantharidin delivery and later incorporated into a dual BR2/anti-CA IX targeting platform (Zhang et al., 2019; Zhang et al., 2017). Other approaches include plasmin-binding tripeptide-decorated stealth liposomes designed to exploit plasmin/plasminogen-activator overexpression in HCC (Calandro et al., 2018), and anti-GPC3/CPP44-modified liposomes that combine antigen recognition with tumor penetration for arsenic trioxide delivery, achieving 1.89-fold higher HepG2 uptake and 63.4% tumor inhibition compared with unmodified liposomes (Lin et al., 2025). In addition, Myrcludex B, an HBV-preS-derived lipopeptide, has been used to exploit NTCP-mediated liver targeting in MMP2-responsive liposomes (Cheng et al., 2022). Together, these examples show that peptide-functionalized liposomes can be tailored to different liver cell populations, HCC-associated receptors, and intracellular delivery requirements.

Across the seven hepatic liposomal systems considered here, the biological rationale for receptor selection is generally well established. In contrast, evidence confirming that the intended ligand density was achieved on the liposome surface is often inconsistent or absent. In many cases, the nominal peptide input is effectively used as a substitute for conjugated surface density, although these represent different quantities. Product identity, conjugation efficiency, residual reactive groups, and the number of ligands per particle each require separate verification. This gap limits reproducibility and makes it difficult to interpret density-dependent biological effects.

Our earlier review of peptide-functionalized hepatic liposomes discussed thiol–maleimide and related conjugation strategies mainly at the level of method category, including pre-insertion versus post-insertion approaches and bond type (Maroof et al., 2026). However, it did not resolve the quantitative relationship between nominal formulation, reactive lipid content, and actual ligand density on the liposome surface. The present Perspective addresses this unresolved issue. Hydrolysis, steric occlusion, bilayer orientation, and thiol re-oxidation are individually well described in the thiol-maleimide literature; what has not been established is how these established phenomena combine, alongside the peptide-side reactive-thiol pool, into a single quantitative accounting that links nominal formulation input to conjugated and biologically accessible ligand density. This integrative accounting, not the identification of the individual mechanisms, is the contribution advanced here. The framework is developed inductively, because the currently available hepatic liposome literature does not directly report maleimide availability or systematically relate it to achieved peptide density.

Rather than providing a systematic review, this Perspective uses a purposive set of hepatic peptide-targeted liposomal systems to examine how ligand conjugation and surface-density information are currently reported. The seven systems were selected from the hepatic peptide-liposome literature surveyed in our earlier review (Maroof et al., 2026) and were limited to studies that used thiol-maleimide chemistry to attach a peptide, or a peptide-based dual ligand, to a PEGylated liposomal surface for hepatic or HCC targeting. Studies were included when they provided sufficient methodological detail, such as stoichiometric ratios, preparation route, reaction conditions, purification strategy, or partial yield information, to allow the reconstruction attempted here. For each system, the extracted information includes the peptide or antibody sequence, liposome formulation, preparation route, reaction conditions, purification method, conjugation-confirmation assay, calculation approach, and exact source location. These details are summarized in Supplementary Table S1. Throughout the Perspective, experimentally reported values are distinguished from values calculated from published data and from cross-study hypotheses introduced for comparative interpretation.

The maleimide-availability problem is not specific to hepatic targeting. Hydrolysis, steric crowding, and thiol re-oxidation are inherent limitations of thiol–maleimide chemistry (Figure 1), regardless of the target tissue, receptor, or ligand used. Hepatic peptide-liposome systems were selected here because they provide a useful and biologically relevant case set in which the consequences of unmeasured surface density can be clearly observed. They also include diverse targets, preparation routes, and research groups. The repeated lack of complete reactive-site reporting across these seven systems therefore points to a broader reporting issue, rather than to formulation-specific shortcomings. For this reason, the reactive-site accounting framework proposed in this Perspective should be relevant to thiol–maleimide-conjugated nanocarriers more generally, with hepatic liposomes serving as a representative example.

**FIGURE 1:**
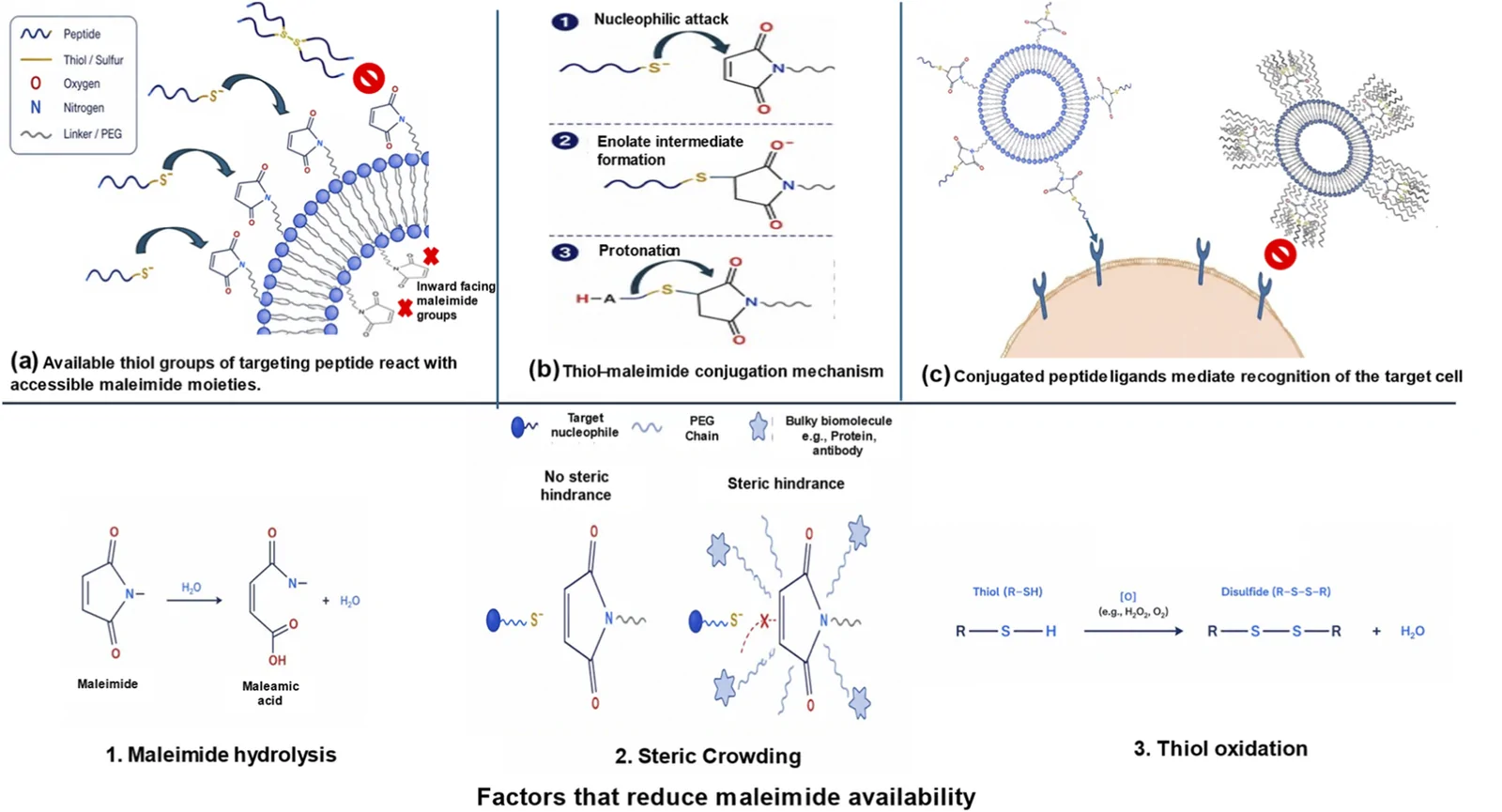
Thiol-maleimide conjugation of targeting peptides to liposomal surfaces and the principal factors that reduce effective maleimide availability. (**(a–c)**, top row) Overview of the conjugation and targeting sequence. **(a)** Cysteine-modified targeting peptides, each bearing a free thiolate (–S^-^), approach a PEGylated lipid bilayer displaying maleimide-functionalized headgroups. Not every thiolate that approaches the bilayer reacts productively (indicated by the blocked/prohibited symbol): a maleimide group may already be hydrolysed, oriented away from the aqueous phase, or sterically inaccessible, so peptide input and successful surface engagement are not equivalent. **(b)** The thiol-maleimide Michael addition mechanism proceeding through three steps. **(c)** Peptide-conjugated liposomes engage cell-surface receptors on the target cell. Conjugation at the liposome surface does not guarantee productive receptor engagement (blocked symbol): a conjugated ligand may be misoriented, sterically occluded by neighbouring PEG or ligands, or simply not encounter a receptor, so conjugated ligand density and biologically accessible ligand density are distinct quantities. (Bottom row) Factors that reduce maleimide availability, considered separately for the maleimide- and peptide-side reactive pools. (1) Maleimide hydrolysis. In aqueous buffer, the maleimide ring can undergo base- or time-dependent hydrolysis to the ring-opened maleamic acid, a product that lacks the electrophilic double bond required for thiol-Michael addition and is therefore unreactive toward peptide thiols. (2) Steric crowding. Schematic comparison of an isolated, unhindered maleimide group (left) against a maleimide group whose approach is obstructed by neighbouring PEG chains and bulky co-conjugated biomolecules such as proteins, antibodies, or other peptides (right, blocked trajectory marked with X). This panel illustrates steric occlusion as a plausible contributing mechanism to reduced conjugation; it is a schematic representation of a proposed mechanism rather than a depiction of experimentally measured conversion, and is presented here as a hypothesis motivating direct measurement rather than as demonstrated evidence that crowding reduces chemical conjugation. **(3)** Thiol oxidation. A free peptide thiol (R–SH) can be oxidised by ambient oxidants (e.g., H_2_O_2_ or dissolved O_2_) to form an intermolecular disulfide (R–S–S–R), consuming two reactive thiols and releasing water. Unlike panels 1 and 2, which affect the maleimide-side reactive pool, this factor depletes the peptide-side reactive-thiol pool and should be considered a separate variable from maleimide availability itself, alongside incomplete reduction/deprotection and peptide solubility under reaction conditions (not depicted).

## Maleimide availability as an underreported determinant of conjugation outcome

Under commonly used coupling conditions (pH 6.5–7.5), a peptide thiolate adds nucleophilically to the maleimide double bond to form a thiosuccinimide adduct (Supplementary Figure S1), typically with high thiol selectivity (Renault et al., 2018). Conversion depends jointly on the concentrations and accessible fractions of both reactive partners, as well as pH, time, temperature, peptide properties, and purification; nominal stoichiometry alone is therefore insufficient to predict the final conjugated density (Kim et al., 2024).

The clearest evidence comes from Calandro et al.’s plasmin-binding tripeptide (TRP) system: using a 1:4 TRP-to-maleimide molar ratio, only 33% of the input TRP was successfully conjugated to the surface (∼300 molecules/liposome). It is the only study in this seven-system case set to quantify conjugation efficiency as a fraction of input (Calandro et al., 2018). Given that Oswald et al. demonstrated a ∼37% loss in surface-accessible maleimide functionality during pre-insertion processing (due to inward lipid orientation and rapid initial hydrolysis) (Oswald et al., 2016), a retention value that cannot be used to numerically adjust Calandro et al.’s nominal 4:1 ratio, because comparable hydrolysis kinetics have not been established for this formulation. Oswald et al.’s retention value was measured in a DMPC/cholesterol liposome system prepared by solvent injection, whereas Calandro et al. used thin-film hydration followed by sonication with a different lipid composition (Calandro et al., 2018). Because the lipid compositions and preparation routes differ, the retention value cannot be transferred directly. This is not a measurement of Calandro et al.’s formulation and does not quantitatively explain the 33% yield; instead, it shows why formulation-specific measurements of accessible maleimide, and reduced peptide thiol are needed before nominal stoichiometry can be interpreted.

Du et al. used a 10:1 liposome:peptide molar ratio (as stated; basis not specified). If calculated on total lipid from the reported composition (EPC/Chol/mPEG-DOPE/MAL-PEG-DOPE, 2:1:0.1:0.02), this corresponds to a nominal Mal:cRGD ratio of approximately 1:16, i.e., peptide in large excess. Conjugation efficiency was not measured (Du et al., 2007); Cheng et al. reported a 3:1 ratio and reported only the mass of conjugated peptide (37.2 μg/mg lipid), not fraction of input (Cheng et al., 2022); Zhang et al. used a 1:1 Mal:BR2 ratio and confirmed product identity by MALDI-TOF without quantifying efficiency (Zhang et al., 2017); and Lin et al.’s dual anti-GPC3/CPP44 system reported neither ratio nor efficiency for either ligand; moreover, the published CPP44 sequence lacks the cysteine stated in the Methods, leaving the source of the peptide’s reactive thiol unresolved (Lin et al., 2025). Within the seven systems reviewed here, MALDI or SDS-PAGE confirmation of product identity is repeatedly used in place of quantitative yield measurement, a pattern worth noting given that nominal surface density is associated, in the same studies, with biological outcome: Zhang et al. found 0.5 mol% BR2 gave uptake indistinguishable from unmodified liposomes, whereas 2 mol% increased uptake (Zhang et al., 2017), and interpretation of Lin et al.’s dual-ligand advantage depends on both ligand densities being adequate simultaneously (Lin et al., 2025). These density-response relationships cannot be reliably interpreted when conjugation efficiency at each nominal density is unknown. Table 1 summarizes stoichiometry, efficiency reporting, and the resulting characterization gap across the systems reviewed here.

**TABLE 1:** Thiol-maleimide conjugation design and reporting across hepatic-targeted liposomal systems.

System/target	Receptor	Nominal mal: peptide ratio	Conjugation efficiency	Key gap	Biological consequence	Ref
cRGD-SSL (Du et al.)	Collagen VI (activated HSC)	10:1 liposome:peptide (as stated); basis not specified; ∼1:16 Mal:peptide if calculated on total lipid	Not reported	Maleimide lipid pre-incorporated during liposome formation; efficiency unmeasured	>10-fold HSC accumulation cannot be linked to a known surface density	Du et al. (2007)
GE11-liposomes (Tang et al.)	EGFR (HCC)	Not reported	Not reported	Calculated at ∼0.38 mol% from reported concentrations; lipid was purchased pre-conjugated, so this reflects input ratio only, with no post-assembly verification of retained or outer-leaflet fraction	Targeting/endocytosis reported, but actual GE11/particle is unknown	Tang et al. (2024)
BR2-liposomes (Zhang et al., 2017)	Gangliosides	∼1:1	Not quantified (MALDI only)	Efficiency at synthesis step unreported	0.5% vs. 2% density-response cannot be tied to actual yield	Zhang et al. (2017)
DF-Lp, BR2+anti-CA IX (Zhang et al., 2019)	Gangliosides + CA IX	1:1 (BR2); Ab not stated	Not reported, either ligand	Two elevated-temp steps risk added hydrolysis	Dual-ligand synergy vs. single-ligand may partly reflect uncharacterized density differences	Zhang et al. (2019)
Dl-ATO-Lp, anti-GPC3+CPP44 (Lin et al., 2025)	GPC3 + HCC membrane	CPP44: maleimide and Ab:maleimide ratios not reported	Not reported, either ligand	Antibody coupling buffer and pH not reported clearly	1.89-fold uptake/63.4% inhibition cannot be attributed to specific densities	Lin et al. (2025)
Tripeptide-TLP (Calandro et al., 2018)	Plasmin/plasminogen receptors	4:1 (Mal:TRP)	33% of TRP input; ∼300 molecules/liposome	Only system with true efficiency data	3–5-fold cytotoxicity gain reported alongside a quantified density; no density–response series	Calandro et al. (2018)
myrB-PPP-LIP (Cheng et al., 2022)	NTCP	3:1 reported; direction not specified	Partial (mass only, no input reported)	Active maleimide, free thiol, and fraction of input conjugated were not measured	Enhanced liver accumulation reported, but batch-to-batch ligand density remains unverifiable	Cheng et al. (2022)

† The GE11-lipid content of ∼0.38 mol% was calculated from the formulation values reported by (Table 1) and was not directly stated by the authors.

The calculation was performed as: GE11-lipid mol% = [n(DSPE-PEG-GE11)/n(total lipid)] × 100 = [0.67 mM/175.17 mM] × 100 ≈ 0.38%. Because DSPE-PEG2000-GE11 was used as a commercially pre-conjugated lipid rather than generated *in situ*, this value reflects the nominal formulation input ratio at mixing, not a post-assembly measurement; the authors report no assay confirming the fraction of this input retained on the finished, purified liposome or its distribution between the outer and inner bilayer leaflets. The calculated value should therefore be interpreted as reconstructed nominal input content, not experimentally verified surface ligand density.

Taken together, the seven systems in Table 1 are a small, purposively selected, and methodologically heterogeneous case set, and most do not directly measure maleimide availability or final ligand density. This evidence base demonstrates deficiencies in reporting and characterization; it does not establish maleimide availability as the primary cause of the differences in conjugation or biological performance observed across these studies. Where a mechanistic link is proposed below, it is offered as a plausible explanation consistent with the reporting gaps identified, not as an experimentally demonstrated effect, and the two should not be conflated. Two studies outside the hepatic set show what more complete reporting looks like. Gray et al. quantified peptide-liposome conjugation directly using a validated fluorescence assay against a peptide standard curve, reporting conjugation efficiencies consistently >90% across three maleimide densities for an αvβ6-targeted, non-hepatic liposomal system, showing that high efficiency can be documented when yield is measured rather than assumed (Gray et al., 2012). Martínez-Jothar et al. also varied preparation method, storage temperature, molar ratio, and reaction time for maleimide-functionalized nanoparticles, and found that, for one peptide ligand, a 2:1 maleimide: thiol ratio gave 84% ± 4% conjugation, whereas a 5:1 ratio with a longer incubation gave 58% ± 12%. The result also shows why no single maleimide: thiol ratio can be transferred across ligands and formulations (Martínez-Jothar et al., 2018).

## Mechanisms that erode effective maleimide availability

### Hydrolysis

Maleimide rings can hydrolyse to non-reactive maleamic acid during aqueous processing, with the extent depending on formulation and process conditions (Niño-Ramírez et al., 2022). Oswald et al. showed that pre-insertion of DSPE-PEG-maleimide during liposome formation retains only 63% of active maleimide after preparation, falling to 32% after dialysis, versus 76% retention with post-insertion of pre-formed micelles (Oswald et al., 2016). The protocols in which maleimide lipid is incorporated during initial liposome formation expose maleimide to aqueous conditions and thermal processing before conjugation. Oswald et al.’s result shows that such processing can substantially reduce the available number of surface-active maleimides, but the exact retention percentage is specific to their DMPC/cholesterol formulation and process (Oswald et al., 2016) and cannot be applied to every formulation reviewed here; indeed it identifies a formulation-specific loss mechanism that should be measured rather than inferred from nominal lipid content.

### Steric crowding

At high ligand densities, steric crowding may reduce access to remaining maleimide sites, but the magnitude of this effect is formulation specific. Zhang et al.’s 0.5%/2% BR2 uptake threshold (Zhang et al., 2017) and Lin et al.’s rougher, irregular TEM morphology at dual-ligand density (Lin et al., 2025) are compatible with crowding-limited chemical conversion, but neither directly demonstrates that crowding reduced conversion itself: differences in uptake at different nominal ligand densities, or morphological changes in dual-ligand formulations, could equally result from changes in receptor engagement, ligand presentation, particle properties, or other downstream phenomena unrelated to chemical conjugation. These examples should therefore be read strictly as hypotheses requiring direct experimental measurement of conversion and ligand accessibility, not as evidence of a steric mechanism. Cheng et al. reported a different accessibility problem: long PEG chains can stabilize circulation while shielding the cell-penetrating peptide (Cheng et al., 2022), requiring enzymatic PEG shedding for activity.

### Thiol re-oxidation and competing reactions

Reduction (TCEP/DTT) or chemical thiolation (e.g., SATA) generates reactive thiols on peptides or antibodies (Chakraborty et al., 2024), and this pool is a variable distinct from maleimide availability itself: delay between thiol generation and conjugation invites re-oxidation, deprotection can be incomplete, and peptide solubility under the reaction buffer can limit the free-thiol fraction actually available to react (Mthembu et al., 2025). None of these peptide-side variables is measured in the seven systems reviewed here. Reaction pH is a further, separate variable affecting the maleimide side: maleimide-thiol selectivity is generally reported over pH 6.5–7.5 (Niño-Ramírez et al., 2022), though a pH difference of 7.4 versus 7.5 alone does not establish that meaningful amine competition or hydrolysis occurred in a given formulation. Cheng et al.’s myrB coupling proceeded at pH 7.4 (Cheng et al., 2022), while Zhang et al.’s BR2 coupling used pH 6.5 (Zhang et al., 2017), the real limitation is that neither study tracks active maleimide or reduced peptide thiol over the course of the reaction, so the coupling yield in either case cannot be attributed to a specific cause.

## A design framework for rational stoichiometric control

This design framework is the central element of the Perspective. It converts the reactive-site accounting terms introduced above into an explicit workflow specifying which parameters should be measured before conjugation, after purification, and during biological characterization, corresponding to the sequence from nominal input, through experimentally measured reactive sites and conjugated density, to biologically accessible density and functional avidity. Throughout this workflow, product-identity assays such as MALDI-TOF and SDS-PAGE can support confirmation that a conjugate has formed, but cannot by themselves establish conjugation efficiency or surface ligand density; the quantitative measurements set out below are required for that purpose, and this distinction is applied consistently when evaluating the individual studies discussed throughout this Perspective.

Before conjugation: quantify active, reactive maleimide using a calibrated thiol-probe assay and quantify reduced, accessible peptide thiol using Ellman’s reagent or a validated fluorescent thiol probe, each normalised to recovered lipid or peptide input rather than nominal input.

After purification: quantify residual free peptide, total conjugated peptide, and total recovered lipid, and use these to calculate conjugation efficiency as a fraction of input and conjugated ligand density as molecules per particle; MALDI-TOF or SDS-PAGE may complement this by confirming conjugate identity but do not substitute for it.

During biological characterization: determine biologically accessible ligand density and functional avidity under assay-relevant conditions, and monitor storage stability and batch-to-batch variation of the conjugation layer, since these are not captured by size or PDI measurements alone.

### Treat reactive-site stoichiometry as a critical formulation variable

It should be explicitly set and reported, alongside size, PDI, encapsulation efficiency, and zeta potential, with the target density set after accounting for measured or experimentally justified loss for the chosen preparation method. Table 1 shows the practical difference: Calandro et al.’s measured 33% efficiency at nominal 4:1 converted a nominal input ratio into an interpretable estimate of ∼300 molecules/liposome (Calandro et al., 2018), while ambiguous or unreported stoichiometry and identity-only confirmation in the other systems leave actual density unknown despite compelling biology.

Operationally, the framework requires two parallel accounting terms, not one. Effective reactive maleimide sites per particle can be represented as nominal maleimide per particle multiplied by an experimentally measured maleimide-availability factor, while the co-equal peptide-side term, available peptide thiol, should be represented as nominal peptide input multiplied by an experimentally measured free-thiol fraction reflecting cysteine oxidation, disulfide formation, incomplete reduction or deprotection, and solubility or handling losses. Because conjugation efficiency is set jointly by whichever reactive pool is limiting under the reaction conditions used, neither term can be estimated from nominal stoichiometry alone, and both should be measured before their product is used to predict conjugated density (Martínez-Jothar et al., 2018). The maleimide-availability factor may be decomposed into outward-facing, unhydrolysed, and sterically accessible contributions, but these terms should not be multiplied as independent fractions unless their dependencies have been established. A practical measurement route is to quantify active maleimide immediately before peptide addition using a thiol-containing probe such as cysteine or glutathione, with the probe concentration calibrated against a freshly prepared maleimide standard curve (McConnell et al., 2020; Moser et al., 2015). An aliquot of the liposome suspension is incubated with a known excess of probe for a defined reaction period under the same buffer and temperature conditions used for conjugation, after which the reaction is quenched by addition of excess cysteine or another validated thiol scavenger (McConnell et al., 2020; Moser et al., 2015). Residual probe can then be quantified by HPLC or a validated colorimetric/fluorometric assay, allowing active maleimide to be calculated from probe consumption. In parallel, the reduced and accessible peptide-thiol pool can be measured immediately before coupling using a thiol-reactive reagent such as Ellman’s reagent (DTNB) or a validated fluorescent thiol probe, with calibration against the corresponding free peptide-thiol standard.

Both measurements should be normalised to recovered lipid rather than to nominal lipid input. Total lipid recovery should therefore be determined independently, for example, from phosphorus or a validated lipid-specific assay (Yang et al., 2025), and used to express active maleimide as nmol per mg recovered lipid and, where particle concentration is known, as molecules per particle. The same lipid-recovery correction should be applied when calculating conjugated peptide density from the residual-free-peptide or total-peptide measurement. These measurements provide a practical distinction between the maleimide-side reactive pool and the reduced, accessible peptide-thiol pool without assuming that either is equivalent to final conjugated ligand density. The resulting sequence of measurements is therefore nominal input, experimentally measured reactive sites, conjugated density, biologically accessible density, and functional avidity.

### Distinguish and evaluate incorporation and coupling routes experimentally

Post-insertion of pre-formed DSPE-PEG-maleimide micelles into pre-extruded liposomes better preserves maleimide reactivity (Oswald et al., 2016). Post-insertion may preserve a higher fraction of outward-facing reactivity in a given formulation, but insertion efficiency depends on liposome size, lipid phase behaviour, PEG-lipid structure, temperature, incubation time, and removal of residual micelles. Route selection should be based on measured maleimide retention, PEG-lipid incorporation, residual micelles, conjugation efficiency, molecules per particle, biologically accessible ligand density, storage stability, and batch-to-batch variation, rather than assumed from the preparation method alone (Zhang et al., 2019; Zhang et al., 2017; Lin et al., 2025).

### Report conjugation efficiency and absolute surface density

Calandro et al. show that this can be done: quantifying free peptide after liposome lysis and subtracting from input allows their cytotoxicity data to be interpreted alongside a reported surface density (Calandro et al., 2018). Neither dual-ligand system (Zhang et al.; Lin et al.) reports this for either component, despite biological synergy that depends on both ligands reaching effective density simultaneously (Zhang et al., 2019; Zhang et al., 2017; Lin et al., 2025). Even where conjugation is quantified at all, the assay itself is not standardised across this literature: Calandro et al. use indirect subtraction of free peptide after liposome lysis, Cheng et al. exploit myrB’s intrinsic tryptophan fluorescence against a peptide standard curve, and the remaining systems rely solely on MALDI-TOF or SDS-PAGE identity confirmation with no quantitative yield measurement at all. These few reported efficiency values are not directly comparable unless each assay is known to capture the same losses, including those during purification and storage. A practical characterisation should therefore include the measured number of accessible maleimides before coupling, reduced free thiol, residual free peptide after purification, total peptide conjugated, total lipid recovery, molecules per particle, storage stability, and, where feasible, single-particle measurements of ligand-density heterogeneity.

## Discussion

Conjugation efficiency, absolute conjugated ligand density, and biologically accessible ligand density answer different questions, and consistently maintaining this distinction is necessary when interpreting the biological results reviewed above. Biological effects such as increased uptake, accumulation, cytotoxicity, or tumor inhibition should not be attributed to ligand density when the actual number of conjugated and accessible ligands per particle was not experimentally determined; where that number is unknown, density can be discussed only as a candidate, unverified explanation. Both conjugation efficiency and absolute surface density are needed to interpret hepatic targeting data. Receptor-binding avidity depends on the absolute number of productive ligand-receptor interactions per particle, not the fraction of input conjugated, so both parameters need reporting to meaningfully interpret uptake, biodistribution, or tumour-inhibition results (Hamzeh et al., 2025; Maslanka Figueroa et al., 2020). This distinction matters particularly for the dual-ligand systems: without knowing actual anti-GPC3/CPP44 or BR2/CA IX densities, apparent synergy cannot be interpreted independently of possible differences in effective density between single- and dual-ligand comparators (Zhang et al., 2019; Zhang et al., 2017; Lin et al., 2025). Residual free ligand, if present after purification, could compound this because free CPP44 and free plasmin-binding tripeptide may retain biological activity independent of conjugation, potentially confounding attribution of effect to the surface-bound fraction (Calandro et al., 2018; Lin et al., 2025).

From a development standpoint, maleimide-related batch variability may be missed by size and PDI measurements alone: consistent size and PDI across storage (reported throughout this literature) does not establish conjugation-layer integrity, which can vary with preparation temperature, extrusion duration, or lipid lot without any detectable change in these standard metrics (Shahiwala, 2025). For dual-ligand products in particular, reactive-site stoichiometry, per-ligand conversion, and accessible ligand density are candidate material attributes, process parameters, and potential critical quality attributes. Their criticality and suitability for specification or release testing should be established through product-specific risk assessment, validated assays, and density-function relationships. This approach is consistent with the FDA’s risk-based Chemistry, Manufacturing, and Controls (CMC) framework for liposomal drug products, in which the identification of material attributes, process parameters, and critical quality attributes is product-specific and supported by scientific evidence, rather than prescribed as a universal requirement (FDA and U. S. F. a. D. A., 2018). Within this framework, reactive-site stoichiometry and related measurements should be evaluated according to their demonstrated impact on product quality and performance, rather than assumed to constitute universal CQAs.

The seven systems reviewed here—cRGD-SSL, GE11-liposomes, BR2-liposomes, BR2/anti-CA IX liposomes, tripeptide-TLP, GPC3/CPP44 liposomes, and myrB-PPP-LIP—provide proof of concept but their results would be easier to interpret and reproduce if reactive sites and ligand density were measured (Du et al., 2007; Tang et al., 2024; Zhang et al., 2019; Zhang et al., 2017; Calandro et al., 2018; Lin et al., 2025). Within the seven systems examined, only Calandro et al. explicitly quantify conjugation efficiency as a fraction of input, which makes the reporting gap in this case set clear. Single-particle methods capable of quantifying particle-to-particle ligand-density distributions are needed to test how density and accessibility jointly affect targeting performance in the hepatic biological milieu. Reactive-site accounting, route-specific validation, and quantitative reporting can be implemented now, before single-particle methods become routine.

## Scope and limitations

This Perspective is limited by its purposive hepatic case set, heterogeneous source reporting, reconstructed stoichiometric ratios, and the absence of formulation-level raw data. The examples identify measurement gaps but do not establish that maleimide availability is universally the dominant determinant of conjugation. Retention values measured in one lipid composition or preparation process should not be transferred to another without validation, and total conjugated ligand should not be equated with receptor-accessible ligand in biological media.

A direct test of the framework would use a factorial design to measure accessible maleimide and reduced peptide thiol while varying their ratio, concentration, pH, time, temperature, PEG architecture, and liposome size. Conjugation efficiency, molecules per particle, accessible ligand density, and receptor-binding activity should be measured as distinct outcomes.

## Data Availability

The original contributions presented in the study are included in the article/Supplementary Material, further inquiries can be directed to the corresponding authors.
