# Supplementary material for: Maleimide availability in thiol-maleimide peptide conjugation on PEGylated liposomes: a reactive-site accounting framework illustrated using hepatic drug-delivery systems
Source: Front Drug Deliv. 2026 Oct 13;6:1964437. doi: 10.3389/fddev.2026.1964437 (PMC13710358; doi:10.3389/fddev.2026.1964437)
Supplement: Supplementary file 1 [file Table1.docx]

**Supplementary Table S1. Extraction Sheet for Seven Hepatic Peptide-Liposome Systems**

This sheet records, for each system reviewed in the main text, the peptide/antibody sequence and mass, liposome formulation, preparation route, reaction conditions, reducing/thiolation agent, purification method, conjugation-confirmation assay, calculation method (where applicable), and exact source location. Values are transcribed directly from the cited primary source; where a value is not stated in the source, this is noted explicitly rather than inferred. Where the source itself is ambiguous (e.g., ratio direction), this is flagged rather than resolved.

**1. cRGD-SSL (Du et al., 2007)**

**Full citation:** Du, S.L., Pan, H., Lu, W.Y., Wang, J., Wu, J. and Wang, J.Y., 2007. Cyclic Arg-Gly-Asp peptide-labeled liposomes for targeting drug therapy of hepatic fibrosis in rats. *The Journal of pharmacology and experimental therapeutics*, *322*(2), pp.560-568.

| **Field** | **Value** | **Source location** |
| --- | --- | --- |
| Peptide sequence | C*GRGDSPK* (Cys-Gly-Arg-Gly-Asp-Ser-Pro-Lys); cyclic via amide bond between Lys and Cys side chains (modified from original C*GRGDSPC*, which cyclized via disulfide) | "Synthesis of cyclic RGD peptide..." section |
| Molecular mass(es) | Not stated | — |
| Formulation | EPC:Chol:mPEG2000-DOPE:MAL-PEG-DOPE = 2:1:0.1:0.02 (molar ratio) | "Synthesis of cyclic RGD peptide..." section |
| Preparation route | Lipid film (chloroform evaporation) → hydration in appropriate buffer (PBS for IFN-loading) → extrusion, 100 nm membrane, 15 passes. Pre-insertion of MAL-PEG-DOPE during film formation. | Same section |
| Reaction conditions | Cyclic RGD peptide incubated with IFN-loaded liposomes at a reported molar ratio of 10:1 overnight; temperature not reported; coupling buffer/pH not reported. (PBS is mentioned only for IFN loading.) | Same section |
| Ratio direction | Ambiguous in source — stated as "peptide at a molar ratio of 10 to 1" without specifying peptide:Mal or Mal:peptide explicitly | Same section |
| Reducing agent | None — thiol supplied via free Cys in synthesized peptide (no natural Cys pair to reduce) | — |
| Purification | Sepharose CL-4B column, twice: (1) to remove free IFN before peptide conjugation, (2) to remove unbound peptide after conjugation | Same section |
| Assay | Not reported — no conjugation efficiency or yield measurement performed | — |
| Calculation | N/A | — |

**2. GE11-liposomes (Tang et al., 2024)**

**Full citation:** Tang, H., Li, L., Wang, B. and Guangxi Scientific Research Center of Traditional Chinese Medicine, 2024. Observation of antitumor mechanism of GE11-modified paclitaxel and curcumin liposomes based on cellular morphology changes. *AAPS Open*, *10*(1), p.1.

| **Field** | **Value** | **Source location** |
| --- | --- | --- |
| Peptide sequence | GE11: YHWYGYTPQNVI (12 aa dodecapeptide) | Abstract / Introduction |
| Molecular mass(es) | Not stated | — |
| Formulation | DSPE-MPEG-GE11 micelle: DSPE-MPEG 3.2 mM : DSPE-MPEG-GE11 0.67 mM; final CUR-PTX@GE11-L: EPC 155.8 mM, CHOL 15.5 mM, DSPE-MPEG-GE11 0.67 mM (Table 1 of source) | Table 1, Methods |
| Preparation route | Two-step microfluidic (INano rapid nanopreparator): (1) CUR-PTX@L liposomes formed separately from DSPE-PEG2000/DSPE-PEG2000-GE11 mixed micelles; (2) liposome phase mixed with micellar phase 1:1 v/v at 5 mL/min total flow. Post-insertion of pre-made GE11-lipid conjugate. | "Preparation of CUR-PTX@GE11-L" |
| Reaction conditions | Not applicable. DSPE-PEG2000-GE11 was purchased commercially from Xi'an Ruixi Biotechnology Co., Ltd.; no peptide-lipid conjugation was performed or described in this study. | Materials section |
| Reducing agent | N/A — conjugation not performed in this paper | — |
| Purification | N/A for conjugation | Methods |
| Assay | Not applicable — no conjugation reaction is traceable in this paper; peptide-lipid coupling occurred upstream at the commercial supplier and is not documented in the accessible literature | — |
| Calculation | N/A | — |

**3. BR2-liposomes (Zhang et al., 2017)**

**Full citation:** Zhang, X., Lin, C., Lu, A., Lin, G., Chen, H., Liu, Q., Yang, Z. and Zhang, H., 2017. Liposomes equipped with cell penetrating peptide BR2 enhances chemotherapeutic effects of cantharidin against hepatocellular carcinoma. *Drug delivery*, *24*(1), pp.986-998.

| **Field** | **Value** | **Source location** |
| --- | --- | --- |
| Peptide sequence | Cys-RAGLQFPVGRLLRRLLR (17 aa + N-terminal Cys) | Materials and methods |
| Molecular mass(es) | DSPE-PEG2000-Mal: theoretical/observed 2982.396 (MALDI); DSPE-PEG2000-BR2: theoretical 5108.496, observed ~5011 (MALDI) | Results, "Synthesis of DSPE-PEG2000-BR2" |
| Formulation | Lp: SPC:DSPE-PEG2000 = 96:4; BR2-Lp: SPC:DSPE-PEG2000:DSPE-PEG2000-BR2 = 96:3.5:0.5 (0.5%) or 96:2:2 (2%) | Methods |
| Preparation route | Ethanol injection; pre-formed DSPE-PEG2000-BR2 conjugate incorporated during liposome assembly (pre-insertion). Conjugation step itself: dried lipid film of DSPE-PEG2000-Mal hydrated in HEPES, added dropwise to BR2 peptide solution. | "Synthesis of DSPE-PEG2000-BR2" |
| Reaction conditions | 1:1 molar ratio (Mal-lipid:BR2); 20 mM HEPES/10 mM EDTA-2Na, pH 6.5; 48 h at RT under N₂; then L-cysteine (10× molar excess vs. maleimide) added, 4 h, to quench unreacted maleimide | Same section |
| Reducing agent | None — BR2 supplied with free Cys already present | — |
| Purification | Dialysis, MWCO 3500, against distilled water, 48 h; lyophilized | Same section |
| Assay | MALDI-TOF-MS — confirms product identity/mass shift only; no efficiency or yield quantification | Same section |
| Calculation | N/A | — |

**4. DF-Lp, BR2 + anti-CA IX (Zhang et al., 2019)**

**Full citation:** Zhang, X., Lin, C., Chan, W., Liu, K., Lu, A., Lin, G., Hu, R., Shi, H., Zhang, H. and Yang, Z., 2019. Dual-functional liposomes with carbonic anhydrase IX antibody and BR2 peptide modification effectively improve intracellular delivery of cantharidin to treat orthotopic hepatocellular carcinoma mice. *Molecules*, *24*(18), p.3332.

| **Field** | **Value** | **Source location** |
| --- | --- | --- |
| Peptide/Ab | BR2: Cys-RAGLQFPVGRLLRRLLR (identical to Zhang 2017). Antibody: anti-CA IX rabbit monoclonal (Abcam), ~150 kDa intact | Materials |
| Molecular mass(es) | Intact antibody migrated at ~150 kDa and the reduced half-antibody at ~75 kDa by SDS-PAGE (apparent molecular weights); no mass spectrometric determination was reported. | Results, Figure 1 |
| Formulation | CA IX-Lp: SPC:DSPE-PEG:DSPE-PEG-Mal = 95.8:3.8:0.4; BR2-Lp: SPC:DSPE-PEG:DSPE-PEG-BR2 = 96:2.0:2.0; DF-Lp: SPC:DSPE-PEG:DSPE-PEG-BR2:DSPE-PEG-Mal = 95.2:1.9:2.0:0.87 | Table 1 |
| Preparation route | BR2: ethanol injection, pre-insertion (same method as Zhang 2017). Anti-CA IX Ab: post-insertion — Ab-conjugated DSPE-PEG-Mal micelle incubated with pre-formed CTD-loaded BR2-liposomes at 60 °C for 2 h. | "Preparation and Characterization of Dual-Functionalized Liposomes" |
| Reaction conditions | Ab reduced with DTT, RT, 90 min (molar ratio not stated); reduced Ab incubated with DSPE-PEG-Mal micelle (hydrated in PBS pH 6.6/0.01 M EDTA, 65 °C, 30 min) overnight at 4 °C with gentle agitation. BR2 conditions as in Zhang 2017 (1:1, HEPES pH 6.5, 48 h RT). | Methods 3.4.1–3.4.2 |
| Reducing agent | DTT — for antibody interchain disulfide reduction (150→75 kDa half-antibody). None for BR2 (pre-existing free Cys). | Methods 3.4.2 |
| Purification | Ethyl acetate extraction (×3) to remove residual DTT after antibody reduction; PD-10/Sepharose CL-4B gel filtration to separate the antibody–DSPE-PEG-Mal conjugate from unconjugated antibody and other low-molecular-weight components before post-insertion. | Methods 3.4.2–3.4.3 |
| Assay | SDS-PAGE with Coomassie staining verified antibody reduction and formation of the half-antibody conjugate qualitatively; MALDI-TOF-MS confirmed formation of the DSPE-PEG-BR2 conjugate. Neither method quantified conjugation efficiency or ligand density. | Results, Figure 1 |
| Calculation | N/A | — |

**5. Dl-ATO-Lp, anti-GPC3 + CPP44 (Lin et al., 2025)**

**Full citation:** Lin, C., Sun, J., Yang, Y., Pan, X., Sun, Y., Sun, B. and Gan, C., 2025. Anti-GPC3 antibody and cell-penetrating peptide CPP44 dual-ligand modified liposomes for targeted delivery of arsenic trioxide in the treatment of hepatocellular carcinoma. *Journal of Drug Targeting*, *33*(6), pp.1004-1013.

| **Field** | **Value** | **Source location** |
| --- | --- | --- |
| Peptide/Ab | CPP44: KRPTMRFRYTWNPMK (sequence printed in the manuscript). The Methods additionally state that CPP44 contained a cysteine residue for maleimide coupling, although this cysteine is absent from the published sequence, representing an internal inconsistency. | Introduction; "Synthesis of DSPE-PEG-MAL-CPP44 conjugate" |
| Molecular mass(es) | Internally inconsistent in source: main text states MALDI-TOF-MS major peak "5042.349, which matched the theoretical calculation"; Figure 3d image annotation shows "5058.625." Neither is labeled as observed vs. theoretical. | Results text vs. Figure 3d |
| Formulation | CPP44-ATO-Lp: HSPC 115 mg, DSPE-PEG2000 5 mg, cholesterol 30 mg, DSPE-PEG-MAL-CPP44 11.32 mg (masses only; molar ratio not given). Dl-ATO-Lp GPC3-conjugate amount for post-insertion not stated as a ratio. | "Preparation of Dl-ATO-Lp" |
| Preparation route | Thin-film/ethanol dissolution, injected into 60 °C aqueous ATO solution; sonicated; extruded 200 nm ×3, 100 nm ×3. Methods describe ethanol dissolution/injection into hot aqueous ATO followed by sonication and extrusion, whereas the Results section refers to the process as "reverse-phase evaporation"; the manuscript uses inconsistent terminology for the preparation method. Dl-ATO-Lp formed by post-insertion of GPC3 conjugate at 60 °C, 1 h. | Methods vs. Results, "Preparation of Dl-ATO-Lp" |
| Reaction conditions | CPP44: 34.77 mg CPP44 + DSPE-PEG-MAL micellar solution, N₂, RT, 48 h; then 10× L-cysteine (20.84 mg) quench, 4 h; dialysis MWCO 3500, 48 h. No molar ratio given — masses only. Ab: SATA (1 mg/4 mL DMSO) added to Ab solution at molar ratio SATA:Ab = 1:10, RT, 30 min; deacetylation RT 2 h; combined with DSPE-PEG-MAL (34.30 mg in 2 mL ethanol, hydrated 400 μL water), overnight 4 °C. The CPP44–DSPE-PEG-MAL and antibody–DSPE-PEG-MAL coupling reactions are described using reagent masses and volumes only; no peptide:maleimide or antibody:maleimide molar ratio is reported. | "Synthesis of DSPE-PEG-MAL-CPP44/GPC3 conjugate" |
| Reducing agent | SATA-based thiolation for antibody (acetyl-protected thiol introduced onto primary amines, then deprotected with hydroxylamine) — not a classical disulfide reduction. None specified for CPP44 beyond the stated pre-existing Cys. | Same section |
| Purification | Desalting column (Ab); dialysis MWCO 3500, 48 h (CPP44 conjugate) | Same section |
| Assay | MALDI-TOF-MS (CPP44 conjugate, identity only); SDS-PAGE + Coomassie (GPC3-Ab conjugate, identity only). No efficiency quantification for either. | Results |
| Calculation | N/A | — |

**6. Tripeptide-TLP (Calandro et al., 2018)**

**Full citation:** Calandro, P., Iovenitti, G., Zamperini, C., Candita, F., Dreassi, E., Chiariello, M., Angelucci, A., Schenone, S., Botta, M. and Mancini, A., 2018. Plasmin-binding tripeptide-decorated liposomes loading pyrazolo [3, 4-d] pyrimidines for targeting hepatocellular carcinoma. *ACS Medicinal Chemistry Letters*, *9*(7), p.646.

| **Field** | **Value** | **Source location** |
| --- | --- | --- |
| Peptide sequence | TRP = D-Ala-Phe-Lys, with synthetic C-terminal linker H₂N–CH₂–CH₂–SH (2-aminoethanethiol) introducing the reactive thiol — tripeptide itself has no natural Cys | Scheme 1–2 |
| Molecular mass(es) | Not given as single value; characterized by LC-MS/MS fragmentation (m/z 276.17, 353.20, 219.11, 206.13, 129.10, 191.12, 100.07) | Figure 2 |
| Formulation | TLP: DPPC:CHOL:DPPE-PEG2000:DSPE-PEG2000-Mal = 2:1:0.08:0.02; LP (no peptide): 2:1:0.1 | Methods |
| Preparation route | Thin-layer evaporation; hydrated in 25 mM HEPES/140 mM NaCl, pH 7.4, 60 °C; downsized by sonication to SUV; filtered through 0.2 μm filters (not membrane extrusion) | Methods |
| Reaction conditions | 1:4 molar ratio (TRP:DSPE-PEG2000-Mal, i.e., 4:1 Mal:TRP); overnight, 4 °C | Methods |
| Reducing agent | Not stated | — |
| Purification | Dialysis to separate uncoupled TRP | Methods |
| Assay | Indirect subtraction — total TRP input minus free TRP recovered after liposome lysis (ethanol), quantified by LC-UV-MS | Methods, Results |
| Calculation | 33% of TRP input bound to liposomal surface; ~300 tripeptide molecules/liposome estimated | Results |

**7. myrB-PPP-LIP (Cheng et al., 2022)**

**Full citation:** Cheng, D., Wen, Z., Chen, H., Lin, S., Zhang, W., Tang, X. and Wu, W., 2022. Hepatocyte-targeting and tumor microenvironment-responsive liposomes for enhanced anti-hepatocarcinoma efficacy. Drug delivery, 29(1), pp.2995-3008.

| **Field** | **Value** | **Source location** |
| --- | --- | --- |
| Peptide sequence | myrB = HBV preS/12–47myr lipopeptide; contains intrinsic tryptophan (used for fluorescence quantification). The peptide reacts with surface maleimide groups, implying the presence of a reactive thiol, but the complete sequence and thiol position are not reported. | Introduction; Methods 2.2.2 |
| Molecular mass(es) | Not given numerically | — |
| Formulation | SPC:Chol:HCPT:DSPE-PEG1k-TAT:Mal-PEG2k-PP-DOPE:DSPE-PEG1k = 120:20:7:2:2:1 | Methods 2.2.1 |
| Preparation route | Lipid film hydration: chloroform dissolution, rotary evaporation 50 °C, vacuum dry 2 h; hydrated 10 mM HEPES pH 6.8, RT, 2 h; sonicated 300 W, 5 min, ice bath; extruded 200 nm ×10 (pre-insertion of Mal-lipid during film formation). myrB conjugated to pre-formed liposome surface afterward (post-insertion of peptide) at RT, 4 h, in PBS. Mal-PEG2k-PP-DOPE itself synthesized via two sequential couplings: MMP2-peptide amine + Mal-PEG2k-NHS (amide bond), then EDC/NHS conjugation to DOPE. | Methods 2.2.1–2.2.2 |
| Reaction conditions | Ratio stated as "3:1" — source does not specify which component is the "3" (Mal or myrB); direction is genuinely ambiguous, not merely uncalculated. PBS, pH 7.4, RT, 4 h. | Methods 2.2.2 |
| Reducing agent | Not stated | — |
| Purification | Sepharose CL4B column (size exclusion) to remove unconjugated myrB | Methods 2.2.2 |
| Assay | Fluorescence spectroscopy exploiting myrB's intrinsic tryptophan (ex 280 nm / em 350 nm) against a peptide standard curve (0.0115–0.092 mg/mL in PBS) | Methods 2.2.3; Results 3.1.2 |
| Calculation | Mass-based quantification: 37.2 ± 2.4 μg myrB per mg lipid. Input myrB quantity not stated, so conjugation efficiency as a fraction of input cannot be calculated from this paper. | Results 3.1.2 |

**Cross-system observations**

1. **Three distinct assay types** are used across the seven systems where any confirmation is attempted: (a) indirect subtraction of free peptide (Calandro), (b) intrinsic-fluorophore quantification against a standard curve (Cheng), and (c) identity-only confirmation via MALDI-TOF or SDS-PAGE with no yield measurement (Zhang 2017, Zhang 2019, Lin 2025, Du 2007 — this last reports no assay at all). Tang 2024 performs no conjugation reaction traceable in the paper.
2. **Ratio direction is ambiguous in the primary source itself** (not just uncalculated) for two systems: Du et al. 2007 ("peptide at a molar ratio of 10 to 1") and Cheng et al. 2022 ("3:1" with no stated numerator identity).
3. **Reducing/thiolation strategy varies structurally**, not just in choice of reagent: BR2 and TRP peptides are ordered/synthesized with a pre-existing or appended free thiol (no reduction step needed); the anti-CA IX antibody (Zhang 2019) undergoes classical DTT-mediated interchain disulfide reduction; the anti-GPC3 antibody (Lin 2025) instead undergoes SATA-based primary-amine thiolation — a mechanistically different route to introducing a reactive thiol, not merely a different reagent choice.
4. **One internal mass discrepancy** was identified within a single source (Lin et al. 2025: 5042.349 vs. 5058.625 for the same conjugate, in text vs. figure) and one internal method-naming inconsistency (same paper: Methods describes an ethanol-injection-style process; Results calls it "reverse-phase evaporation").

 **Supplementary Figure S1. Mechanism of thiol-maleimide Michael addition for peptide conjugation to PEGylated liposomal surfaces.**

At pH 6.5–7.5, only a fraction of a typical cysteine side chain is present as the thiolate (S⁻), but this more nucleophilic species reacts selectively with maleimide. The reaction proceeds through (1) thiolate attack at the electrophilic β-carbon of the maleimide double bond, (2) formation of an enolate intermediate, and (3) protonation to yield a thiosuccinimide adduct. The initial adduct should not be described as unconditionally stable: depending on linker structure and medium, it can undergo retro-Michael thiol exchange, whereas succinimide-ring hydrolysis can increase stability.^12^ The wavy blue line represents the targeting peptide; the grey wavy line represents the PEG linker tethering maleimide to DSPE-PEG(2000).
